# China’s provincial process CO_2_ emissions from cement production during 1993–2019

**DOI:** 10.1038/s41597-022-01270-0

**Published:** 2022-04-12

**Authors:** Shiming Liao, Dong Wang, Changyou Xia, Jie Tang

**Affiliations:** 1 School of Economics and Management, Harbin Institute of Technology, Shenzhen, 518055 China; 2 China Resources Environmental Protection, China Resources, Shenzhen, 518000 China; 3 UK-China (Guangdong) CCUS Centre, China Energy Engineering Group Guangdong Electric Power Design Institute, Guangzhou, 510700 China

**Keywords:** Environmental impact, Climate-change mitigation, Governance, Climate change, Environmental impact

## Abstract

Carbon dioxide (CO_2_) emissions from China’s cement production process have increased rapidly in recent decades, comprising the second-largest source of CO_2_ emissions in the country, next only to fossil fuel combustion. However, there used to lack high-quality data to estimate provincial process-related CO_2_ emissions from the cement industry of China. It has been recognised that many previous publications have adopted cement-based accounting methods or national average emission factors to estimate them. This study assembles fundamental provincial clinker production data and provincial clinker emission factors from multiple official statistics sources, following the Intergovernmental Panel on Climate Change (IPCC) methodology (A clinker-based estimation methodology), to develop a high-quality panel dataset of China’s provincial process-related cement emissions during 1993–2019. In 2019, the gross cement process emissions of China amounted to 818.2 Mt CO_2_, and the cumulative emissions between 1993–2019 were estimated to be approximately 12.5 Gt CO_2_. There are significant differences in provincial process-related CO_2_ emissions. The dataset is crucial to provincial cement process emission characterisation and emissions reduction policy-making in China.

## Abstract (summary)

**Table:** 

Measurement(s)	carbon dioxide emission process
Technology Type(s)	computational modeling technique
Factor Type(s)	cement production • clinker production • the emission factor of clinker
Sample Characteristic - Organism	cement • clinker • Nonmetallic Minerals
Sample Characteristic - Environment	manufacturing process
Sample Characteristic - Location	Province (China)

## Background & Summary

As a common building material, cement is widely used in housing and road construction. In general, the global cement industry accounts for about 5–7% of the world’s CO_2_ emissions^1^. Since 1990, global cement production has increased nearly fourfold, the growth rate of which is substantially faster than energy production over the same time period^2^. Global cement production in 2019 is estimated at approximately 4.1 Gt^3^. According to China National Bureau of Statistics, in 2019, China produced 2.3 Gt cement^4^, accounting for 56.8% of the world’s total cement production, and in recent years its process-related emissions from cement production accounted for more than 50% of the global process emissions from cement production^2^. Hence, China’s cement industry is a crucial sector for reducing industrial process CO_2_ emissions in the world. In May 2021, seven ministries of China, including the State Administration for Market Regulation, the Ministry of Industry and Information Technology, the National Development and Reform Commission, and the Ministry of Ecology and Environment, jointly issued the *Opinions on Improving the Quality of Cement Products and Regulating the Cement Market*, which stated that the carbon emissions of the cement industry must reach its peak before 2030^5^. Although it is very important to reduce CO_2_ emissions from the direct combustion of fossil fuels and the use of fossil energy-based electricity in the cement industry, reducing process-related emissions from cement production is also a crucial part of achieving the carbon peaking and neutrality goal of China’s cement industry.

To quantify CO_2_ emissions from the cement production process is the basis of managing process-related CO_2_ emissions from China’s cement production plants. Currently, there lacks of consistency in the reported CO_2_ emissions from China’s cement production process. There are also no official cement process emissions data in consecutive years’ order, with only national-level data for a few years 1994^6^, 2005^7^, 2010^8^, 2012^9^, 2014^10^ being publicly available. Although some studies have estimated process CO_2_ emissions from China’s cement industry, these works rely on the national-level data, such as Emissions Database for Global Atmospheric Research (EDGAR), Carbon Dioxide Information Analysis Center (CDIAC) and Global Carbon Budget (GCB)^11–14^ (Table 1). The more precise provincial-level emission data were seldomly counted. On the other hand, many of these studies calculated the cement-related process emissions based on the cement output^12,15–17^ (that is, using an average cement emission factor to directly multiply with cement output). With the average cement emission factor (0.2906 tonne CO_2_ per tonne cement), Carbon Emission Accounts and Datasets (CEADs) calculated provincial process cement emissions with provincial cement production data during 1997–2019^16,18,19^. However, according to the 2006 IPCC Guidelines for National Greenhouse Gas Inventories^20^, the cement-based method doesn’t reflect the actual process of emission occurrence in the cement production. Those calculations are generally of low accuracy since it does not consider regional differences in cement manufacturing process and cement–clinker ratios across China^21^. The clinker production provides the best activity data for the process emissions calculation of cement industry^20,22^. Only a few studies have recently used the clinker production method to estimate China’s provincial carbon emissions of the cement production process^21^. For example, CEADs used a national average emission factor of 0.4964 tonne CO_2_ per tonne clinker to calculate provincial process cement emissions during 1996–2016^21^. However, in terms of emission factors, those studies use a single average clinker emission factor to calculate the cement emissions of different provinces, which fail to conform to the best practices recommended by the IPCC guidelines^20^. This is because the emission factors of cement industries in different provinces in China are quite different^23^.

**Table 1:** Representative studies of China process-related CO_2_ emissions from cement production.

Data level	References	Institute	Period	Method	Emission factor level
National	Gilfillan *et al*.^12^	Carbon Dioxide Information Analysis Center (CDIAC)	1928–2017	Cement	National
	Crippa *et al*.^14^	Emissions Database for Global Atmospheric Research (EDGAR) 6.0	1970–2018	Clinker	National
	Andrew^11^	Global Carbon Budget (GCB)	1990–2020	Clinker	National
Provincial	Shan *et al*.^16,18^ and Guan *et al*.^19^	Carbon Emission Accounts and Datasets (CEADs)	1997–2019	Cement	National
	Shan *et al*.^21^	Carbon Emission Accounts and Datasets (CEADs)	1996–2016	Clinker	National
	This study	—	1993–2019	Clinker	Provincial

**Fig. 1:**
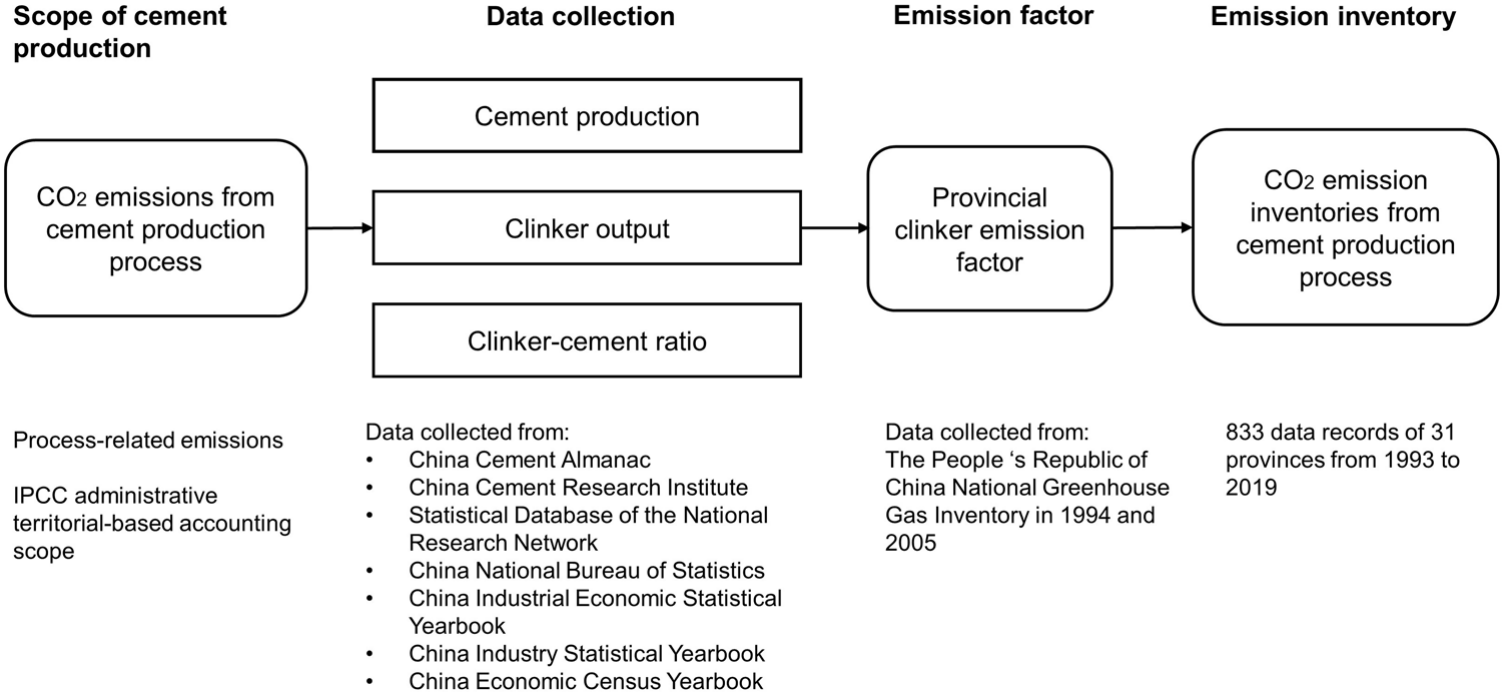
Construction flows of China’s provincial CO_2_ emission inventory from the cement production process.

To provide high-quality data to estimate China’s process-related emissions from cement production, this study developed a panel dataset for the cement industries of China’s 31 provinces during the time interval of 1993–2019. A clinker-based estimation methodology was adopted. The dataset was assembled from provincial-level data of cement production, clinker production and cement–clinker ratios, as well as other basic data and the official provincial emission factors. The dataset provides a robust scientific support for further analyses of China’s greenhouse gas emission issues and emission management strategies. Figure 1 shows the overall structural design of the cement emission estimation methodology in this study, and the dataset is available online at 10.11922/sciencedb.00024.

## Methods

The process-related CO_2_ emissions from cement production in the dataset were estimated according to the IPCC territorial-based accounting scope. It means that these emissions ‘taking place within national (including administered) territories and offshore areas over which the country has jurisdiction’^24^. The administrative territorial accounting method can be used to estimate the human-induced emissions directly within one region’s boundaries^25^.

### Research scope

Carbon dioxide emissions in the cement production process mainly occur in the clinker production process. Clinker is an intermediate product in the production of cement. In the production of clinker, limestone containing calcium carbonate and magnesium carbonate is heated over 1000 °C to calcine the limestone, and carbon dioxide is released as a by-product. In general, there are two primary sources of CO_2_ emission in the cement manufacturing process. The first source of the CO_2_ emission is from the chemical reaction of limestone calcination process (largely *CaCO*_3_ in limestone)^26^, i.e. the process depicted by Eq. (1). The second source is from the direct usage of fossil fuels and electricity that power the cement manufacturing process. In this study, we only focus on CO_2_ emissions from the limestone calcination process. This is because, in CO_2_ emission inventories, fossil fuel and electricity emissions consumed by cement production are normally attributed to the emissions of the energy sector^27^.

1 $$\begin{array}{c}CaC{O}_{3}+{\rm{heat}}\to CaO+C{O}_{2}\\ MgC{O}_{3}+{\rm{heat}}\to MgO+C{O}_{2}\end{array}$$

### Calculate process-related CO_2_ emissions of cement manufacture

According to the 2*006 IPCC Guidelines for National Greenhouse Gas Inventories*, there are three primary methods to measure CO_2_ emissions from cement production process^20^.The first method is to multiply the estimated clinker production by the emission factor. Clinker production estimates are inferred from the output and clinker content ratio of cement by types; exports and imports of clinker are also considered. The second method is to multiply the actual clinker output by the emission factor and the cement kiln dust correction factor. Compared with the first method, the emission accounting method that directly uses clinker production data has lower uncertainty. The third method is to calculate CO_2_ emissions from the cement production process based on the weight and composition of carbonates in raw materials and fuel sources, the emission factor of carbonate, and the proportion of calcination achieved. The third method generally covers a wide range of data and is more accurate. But in fact, it is often difficult to collect plant-level supporting data.

Based on the collected and estimated data of clinker production by province, this paper uses the second method in order to obtain the provincial cement production process emission data from 1993 to 2019. Clinker or cement production data are used, depending on data availability, to estimate the CO_2_ emissions. Following the *2006 IPCC Guidelines for National Greenhouse Gas Inventories*(vol.3 ch.2 p.2.10) and the *2005 China Greenhouse Gas Emissions Inventory Study *(p.103)^20,28^, the calculation process of the second method follows Eq. (2).

2 $$C{O}_{2}\;{\rm{Emissions}}={M}_{cl}\times E{F}_{cl}\times C{F}_{ckd}$$

Where *CO*_*2*_ *Emissions* refer to the emissions of CO_2_ from cement production process, *M*_*cl*_ the weight (mass) of clinker produced, *EF*_*cl*_ the emission factor of clinker, *CF*_*ckd*_ the emission correction factor of cement kiln dust (CKD).

According to chemical reaction formula as in Eq. (1), the emission factor for clinker is determined by Eq. (3):

3 $$E{F}_{cl}={C}_{CaO}\times \frac{44.0095}{56.0774}+{C}_{MgO}\times \frac{44.0095}{40.3044}$$

Where *C*_*CaO*_ is the content of *CaO* in clinker, *C*_*MgO*_ the content of *MgO* in clinker. We directly used the calculated outcome of clinker emission factors from the books about the 1994 and 2005 China Greenhouse Gas Emissions Inventory Study^28,29^.

If there are clinker production data of 31 provinces in the corresponding year, we calculate the CO_2_ emissions of cement production process directly. However, if the clinker production data of year *t* is missing, then the annual clinker data can be estimated with the cement production data by Eq. (4) as below.

4 $${M}_{cl,t}={M}_{c,t}\times \left(\frac{{M}_{cl,t1}}{{M}_{c,t1}}+\frac{{M}_{cl,t2}}{{M}_{c,t2}}\right)\times \frac{1}{2}$$

Where *M*_*cl,t*_ represents the estimated cement clinker output of the missing year *t*, *M*_*c,t*_ the cement production of year *t* without cement clinker data, *t*1 and *t*2 are the latest years with data before and after the data-missing year respectively.

### Emission coefficient

Various organisations have suggested default emission factors for clinker. IPCC Tier 2 and Cement Sustainability Initiative (CSI), respectively, provided emission factors of 0.510 tonne CO_2_ per tonne clinker (not including a correction for CKD) and 0.525 tonne CO_2_ per tonne clinker (including a correction for *MgCO*_3_)^20,30^. Using the cement-based method, EDGAR, CDIAC and CEADs suggested emission factors of 0.390 tonne CO_2_ per tonne cement, 0.499 tonne CO_2_ per tonne cement and 0.2906 tonne CO_2_ per tonne cement, respectively^31^.

*1994 China Greenhouse Gas Emissions Inventory Study*^29^ suggests that the average clinker emission factors of China in 1994 is 0.5277 tonne CO_2_ per tonne clinker. The book also provided the clinker emission factors of various provinces in China in 1994. According to the data, with the exception of a few provinces, the numerical deviations of the clinker emission factors in most of Chinese provinces were relatively small. Shanxi, Liaoning and Hunan have higher clinker emission factors due to higher content of *MgO* in the produced clinker; Ningxia has a low content of *CaO* and moderate content of *MgO* in the clinker; therefore, its clinker emission factor is the lowest. For the calculation of emissions from the cement production process in various provinces from 1993 to 1999, this study uses the clinker emission factors of each province in 1994.

From 2000 to 2019, we adopted the clinker emission factors of each province in 2005 to calculate the provincial process emissions. The 2005 clinker emission factors of provinces and regions in China were given in page 160–161 of the *2005 China Greenhouse Gas Emissions Inventory Study*^28^. However, there are a few provinces where the emission factors are missing in 2005. We assume that the content of $CaO$ and $MgO$ in the clinker of a province is close to the average of the region. Therefore, the missing emission factor per unit of cement clinker can be replaced by the average level of the regions where it is located. The 2005 clinker emission factors of Tianjin, Shanxi and Inner Mongolia were set to the average emission factor of the North China, i.e. 0.5270. The 2005 clinker emission factors of Jilin and Heilongjiang were set to the average emission factor of the Northeastern China, i.e. 0.5458. The 2005 clinker emission factors of Shanghai, Jiangsu, Anhui and Jiangxi were set to the average emission factor of the Eastern China, i.e. 0.5381. The 2005 clinker emission factors of Henan, Hunan and Guangxi were set to the average emission factor of the Central-southern China, i.e. 0.5456. The 2005 clinker emission factors of Chongqing, Guizhou and Tibet were set to the average emission factor of the Southwestern China, i.e. 0.5283. The 2005 clinker emission factors of Qinghai and Shaanxi were set to the average emission factor of the Northwestern China, i.e. 0.5393. The final provincial clinker emission factors are illustrated in Table 2.

**Table 2:** Clinker emission factors of 31 provinces of China in 1994 and 2005 (tonne CO_2_ per tonne clinker).

ID	Province	1994 Clinker EF	2005 Clinker EF
1	Beijing	0.5222	0.5381
2	Tianjin	0.5249	0.5270
3	Hebei	0.5249	0.5269
4	Shanxi	0.5427	0.5270
5	Inner Mongolia	0.5205	0.5270
6	Liaoning	0.5352	0.5458
7	Jilin	0.5223	0.5458
8	Heilongjiang	0.5220	0.5458
9	Shanghai	0.5286	0.5381
10	Jiangsu	0.5286	0.5381
11	Zhejiang	0.5301	0.5326
12	Anhui	0.5261	0.5381
13	Fujian	0.5230	0.5326
14	Jiangxi	0.5219	0.5381
15	Shandong	0.5311	0.5429
16	Henan	0.5262	0.5456
17	Hubei	0.5340	0.5371
18	Hunan	0.5276	0.5456
19	Guangdong	0.5242	0.5488
20	Guangxi	0.5256	0.5456
21	Hainan	0.5306	0.5456
22	Chongqing	0.5283	0.5283
23	Sichuan	0.5264	0.5232
24	Guizhou	0.5282	0.5283
25	Yunnan	0.5278	0.5357
26	Tibet	0.5283	0.5283
27	Shaanxi	0.5294	0.5393
28	Gansu	0.5270	0.5421
29	Qinghai	0.5270	0.5393
30	Ningxia	0.5115	0.5556
31	Xinjiang	0.5231	0.5271

According to the *2005 China national greenhouse gas inventory Study*^28^, the clinker emission factors of 31 provinces in China ranged between 0.5232–0.5556 tonne CO_2_ per tonne clinker, which is higher than 0.4964 tonne CO_2_ per tonne clinker production used by Shan *et al*.^21^. Moreover, provincial differences of clinker emission factors were ignored by these studies, which simply use a national average emission factor to calculate emissions from the cement production process in different provinces. It would cause inaccurate accounting of cement process CO_2_ emissions at the provincial level.

If assuming that the calcined CKD in the system is not lost, then the CKD correction factor will be 1 (Vol.3 ch.2 p.2.12)^20^. According to the *2005 China National Greenhouse Gas Inventory* research group’s field survey and expert experience, Chinese companies directly screen raw material ores, maximise the use of low-grade ores and rocks, and install dust removal devices, so that the amount of unrecovered clinker dust is close to zero (p. 105)^28^. As a result, the correction factor for cement kiln dust would be 1^28^. The uncertainty caused by this way will be considered in the uncertainty analysis later.

### Data source

Up-to-date statistics and analyses of raw materials inputs and clinker production and country-specific emission factors are preferred for estimating the process CO_2_ emissions from cement production^32^. Following this suggestion, our dataset mainly includes two groups of raw data: provincial clinker production data and provincial cement production data. The China National Bureau of Statistics had provided national and provincial cement production data from 1993 to 2019^4^. The provincial clinker production data have different sources in various years. The provincial cement clinker output data in 1993, 1994, and 1997 are respectively from *the 1994 China Industry Economy Statistical Yearbook*, *the 1994 China Greenhouse Gas Emissions Inventory Study*, and *the 1998 China Industry Economy Statistical Yearbook*^29,33,34^. The statistical database of the National Research Network released the provincial cement clinker data for 2002, 2015 and 2016^35,36^. The 2015 provincial clinker output data only includes the first 10 months. This study multiplies the numbers by 1.2 to estimate the entire year’s provincial clinker output. The provincial clinker production data from 2005 to 2007 are from the cement yearbooks by the China Cement Association^37–39^.The provincial cement clinker output data from 2008 to 2014 are respectively from *the 2009 China Industry Economy Statistical Yearbook*, *the 2010 China Industry Economy Statistical Yearbook*, *the 2011 China Industry Economy Statistical Yearbook*, *the 2012 China Industry Economy Statistical Yearbook*, *the 2013 China Industry Statistical Yearbook*, *the 2014 China Industry Statistical Yearbook and the 2015 China Industry Statistical Yearbook*^40–46^. The provincial clinker output in 2017 is mainly based on the 2017 and 2018 economic operation report of China’s cement industry by China Cement Association^47,48^, except the Jiangsu and Zhejiang Province. The provincial cement clinker output data of year 2018 was collected from *the Secondary Industry Volume of the 2018 China Economic Census Yearbook*^49^. The provincial clinker production data of year 2019 is given by the China Cement Association. Most of the provincial clinker data for the remaining years (i.e. years 1995, 1996, 1998–2001, 2003, 2004.) are estimated based on the cement–clinker ratio values of the previous and subsequent years and the current year’s cement production. However, the clinker data of a few provinces in these remaining years are from public sources. For example, the 2004 clinker production of 17 provinces, i.e. Beijing, Shanxi, Inner Mongolia, Liaoning, Shanghai, Zhejiang, Fujian, Jiangxi, Shandong, Hubei, Hainan, Chongqing, Guizhou, Yunnan, Shaanxi, Gansu and Xinjiang, are from the their own 2004 Economic Census Yearbook^50–66^. The 2004 clinker production of Anhui Province is from *the 2005 Anhui Industry Economy Statistical Yearbook*^67^. The 1998 clinker production of Qinghai is from *the 1999 Qinghai Statistical Yearbook*^68^. The 1996 clinker production of Anhui is from *the 1998 Anhui Statistical Yearbook*^69^. The 1995 and 1996 clinker production of Xinjiang are from *the 1997 Xinjiang Statistical Yearbook*^70^. The 1995 clinker production of Beijing, Shanxi, Liaoning, Jiangsu, Zhejiang, and Fujian is from the their own third national industrial census of China in 1995^71–76^.

## Data Records

Our dataset is an Excel file containing six sheets. The six sheets involve the raw cement production, the raw clinker production, the calculated clinker-cement ratio, the clinker emission factor, the final cement process emission inventory data and the uncertainty analysis. The China’s provincial cement production process emissions dataset includes 833 data records (31 × 27 − 4 = 833). The Chongqing city was separated from the Sichuan Province in 1997 and became a municipality directly under the Central Government. Therefore, there is no data for Chongqing during 1993–1996. The annual cement production data for all other provinces of China have been provided in the dataset. Our dataset is stored on the website of Science Data Bank website^77^. It is accessible through a doi link 10.11922/sciencedb.00024.

**Fig. 2:**
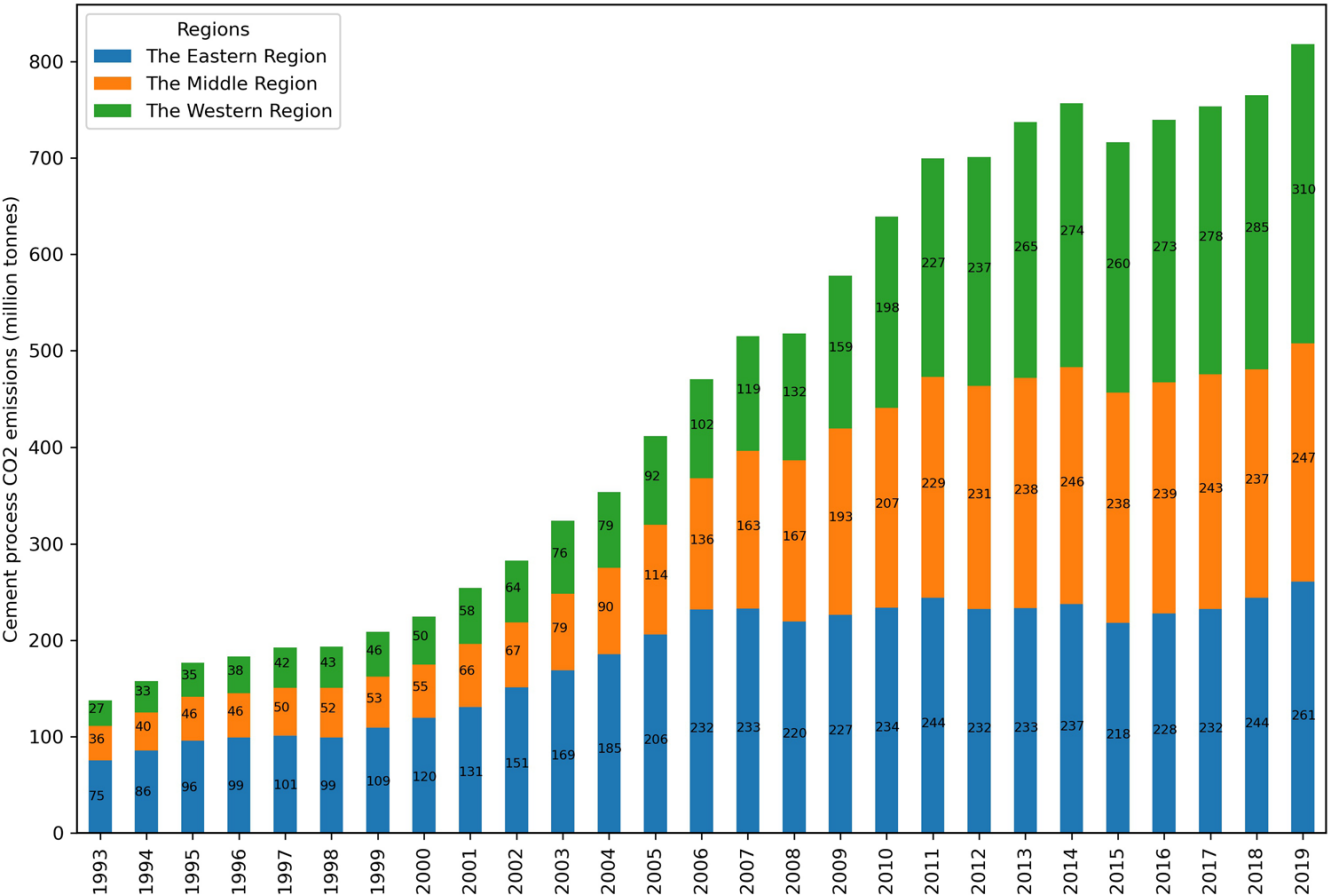
China process cement emissions by regions, 1993–2019. The stack area chart represents CO_2_ emissions from three major economic regions. The Eastern China includes Beijing, Tianjin, Hebei, Liaoning, Shanghai, Jiangsu, Zhejiang, Fujian, Shandong, Guangdong, and Hainan. The Middle China includes Shanxi, Jilin, Heilongjiang, Anhui, Jiangxi, Henan, Hubei, and Hunan. The Western China includes Inner Mongolia, Guangxi, Chongqing, Sichuan, Guizhou, Yunnan, Tibet, Shaanxi, Gansu, Qinghai, Ningxia, and Xinjiang.

Based on the level of economic development, the Mainland China can be classified into three geographic areas, i.e. the eastern, central and western regions. Figure 2 is an illustration of the cement-related emissions of the three major economic regions across China. The figure shows that, in general, China’s cement production process emissions increased steadily during 1993–2000, and began to grow rapidly after 2000. After reaching 757.0 Mt CO_2_ in 2014, it decreased to 716.4 Mt CO_2_ in 2015, and then turned over to reach 818.2 Mt CO_2_ in 2019. The cement emissions in central and western regions have increased substantially since 2006, while the eastern region has basically maintained CO_2_ emissions around 230 Mt CO_2_. The data reflect the large-scale on-going industrialization and construction process in middle and western China.

**Fig. 3:**
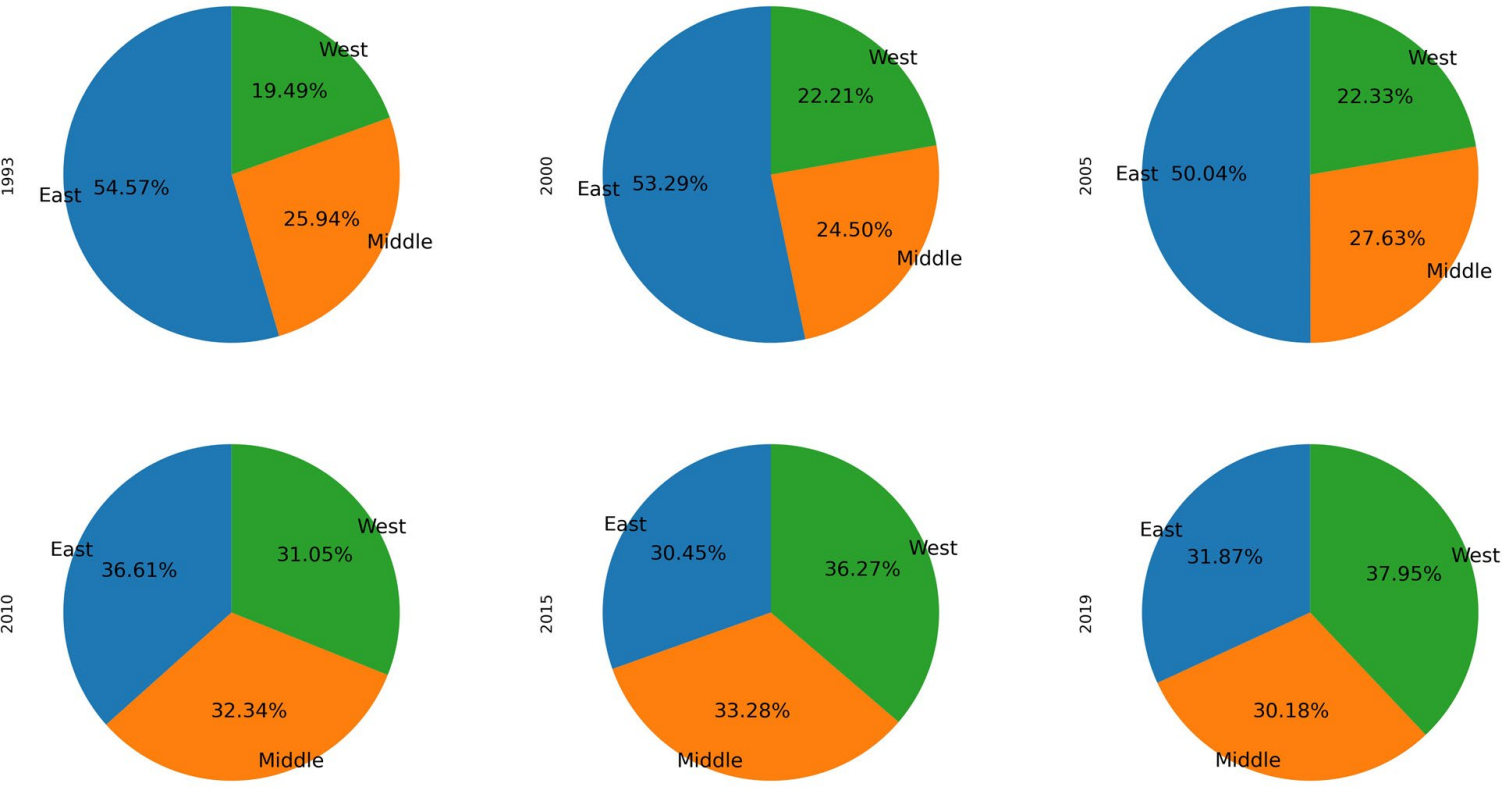
China’s regional structure of process emissions from cement production in 1993, 2000, 2005, 2010, 2015, and 2019.

Figure 3 is an illustration for the proportion of emissions from different regions in 1993, 2000, 2005, 2010, 2015 and 2019. The proportion of emissions in the eastern region decreased from 54.6% in 1993 to 31.9% in 2019; while the central and western regions increased from 25.9% and 19.5% in 1993, to 30.2% and 37.9% in 2019, respectively. At present, each of the eastern, central and western regions accounts for about one-third of the CO_2_ emissions, with the western region being the highest.

**Fig. 4:**
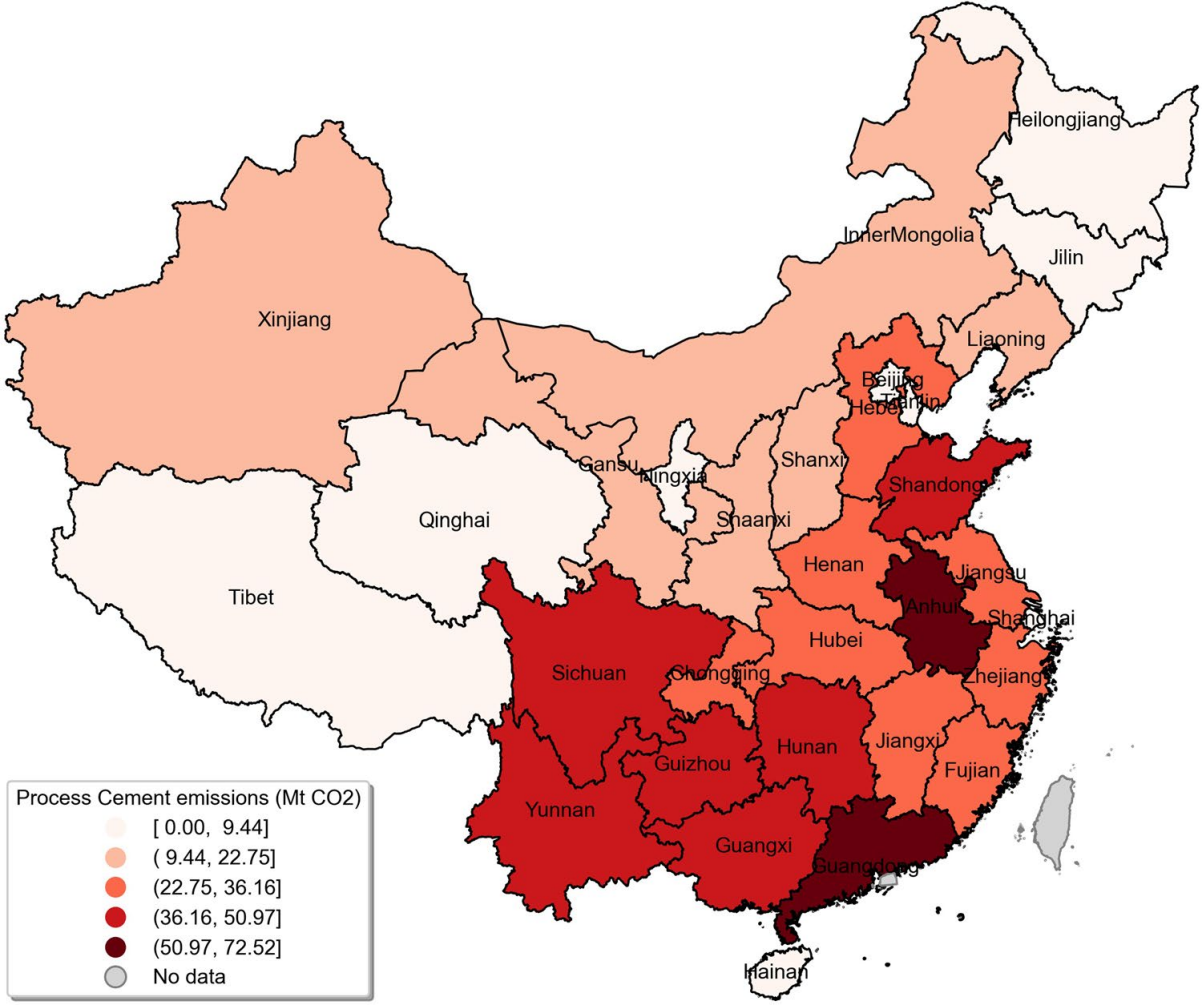
Process emissions from cement production by provinces in 2019 (Mt CO_2_).

Figure 4 shows the current status of CO_2_ emissions from the cement production process of various provinces in China in 2019. It shows that Anhui and Guangdong provinces have the highest process cement emissions, at 72.5 Mt CO_2_ and 59.8 Mt CO_2_ respectively, while Sichuan, Yunnan, Guizhou, Shandong, Guangxi, and Hunan are in the second echelon, with emissions varying from 39.3 to 50.8 Mt CO_2_. The provinces around Anhui, including Jiangsu, Zhejiang, Fujian, Jiangxi, Hubei, Chongqing, Henan and Hebei, are in the third echelon, with emissions ranging from 28.6 to 36.2 Mt CO_2_. The fourth echelon includes Liaoning, Shanxi, Shaanxi, Gansu, Xinjiang and Inner Mongolia, which have emissions between 16.3 and 22.8 Mt CO_2_. Jilin, Heilongjiang, Qinghai, Tibet, Ningxia, Shanghai, Beijing, and Hainan have the lowest emissions levels, which are below 9.4 Mt CO_2_. Table 3 lists provincial emission data for some specific years, including 1993, 2000, 2005, 2010, 2015, and 2019. Shanghai’s cement process carbon emissions have dropped to zero from 2016, and Beijing’s cement process carbon emissions in 2019 have become lower than 1993. The period of the fastest increase in carbon emissions from China’s cement process is mainly between 2000 and 2010.

**Table 3:** Provincial process emissions from cement production in 1993, 2000, 2005, 2010, 2015, and 2019 (Mt CO_2_).

Provinces	1993	2000	2005	2010	2015	2019
Beijing	2.20	2.29	4.13	3.93	2.24	1.38
Tianjin	0.36	0.61	1.27	1.00	0.53	0.39
Hebei	5.93	13.16	22.11	24.95	26.46	34.97
Shanxi	2.57	3.69	8.01	11.69	12.00	19.25
Inner Mongolia	0.80	2.08	6.13	16.37	14.85	18.66
Liaoning	6.07	6.25	9.29	17.61	14.36	22.61
Jilin	2.30	2.82	8.45	14.41	14.41	9.20
Heilongjiang	2.30	3.35	4.73	8.03	6.81	5.90
Shanghai	1.10	0.96	1.90	0.31	0.18	0.00
Jiangsu	11.08	18.91	29.69	30.88	28.26	30.46
Zhejiang	8.00	16.56	34.92	31.44	28.08	30.63
Anhui	6.11	6.77	27.45	54.49	72.68	72.52
Fujian	4.23	6.41	12.16	23.22	24.83	28.56
Jiangxi	3.16	6.00	13.22	23.87	29.13	36.16
Shandong	16.89	27.08	53.38	50.68	42.16	44.08
Henan	8.80	14.18	24.07	32.70	41.96	30.35
Hubei	3.71	7.55	13.13	29.34	27.03	34.26
Hunan	6.80	10.65	14.68	32.17	34.39	39.32
Guangdong	19.16	26.04	35.32	45.77	44.33	59.79
Guangxi	6.37	9.68	14.79	30.79	39.08	41.84
Hainan	0.18	1.38	1.81	4.25	6.70	7.93
Chongqing	0.00	5.23	9.60	17.53	25.81	29.36
Sichuan	7.10	9.66	17.14	45.56	42.09	50.97
Guizhou	1.96	3.47	7.13	15.30	30.46	44.78
Yunnan	3.12	6.83	11.99	21.37	32.42	49.08
Tibet	0.05	0.16	0.48	1.09	1.89	3.10
Shaanxi	2.63	3.50	9.35	19.89	26.21	22.75
Gansu	2.34	3.62	6.24	9.75	17.93	19.13
Qinghai	0.36	0.57	1.29	2.82	6.37	5.10
Ningxia	0.50	1.37	2.82	5.93	6.49	9.44
Xinjiang	1.62	3.69	4.95	12.05	16.21	16.28
Total	176.65	224.52	411.62	639.22	716.38	818.22

**Fig. 5:**
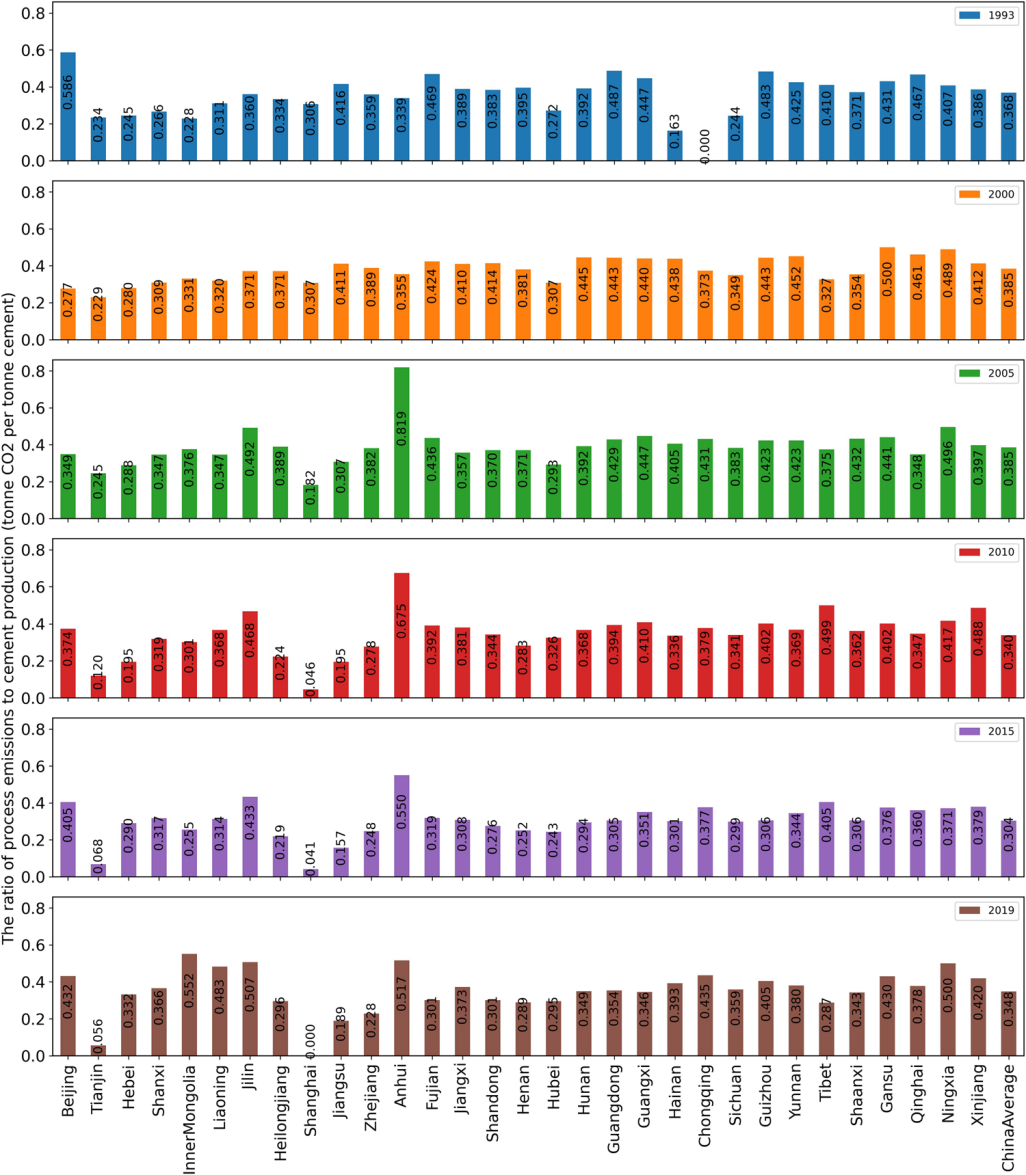
The ratio of process emissions to cement production in 1993, 2000, 2005, 2010, 2015, and 2019 (tonne CO_2_ per tonne cement).

Figure 5 shows the differences in the ratio of process CO_2_ emissions to cement production between provinces in 1993, 2000, 2005, 2010, 2015, and 2019. Cement process carbon emissions per unit of cement production vary significantly among different provinces. It also varies significantly each year from 1993 to 2019. Domestic clinker trade between provinces and international trade between provinces and other countries are important reasons for this difference.

Notably, the ratios of clinker production to cement production in the data set vary by province. For example, it is relatively high (0.9614 in 2019) in Anhui, while the clinker-cement ratio in Jiangsu is relatively low (0.3522 in 2019). Anhui has a large clinker and cement production capacity, and lots of clinker are produced in the cities of Anhui province, such as Wuhu, Tongling, Fanchang, Zongyang and other places along the Yangtze River. Hence, a large part of Anhui’s clinker production is transported to coastal areas downstream the Yangtze River, such as in Jiangsu and Zhejiang, for grinding and processing to produce cement. Clinker trade is one of the important reasons for the difference in the ratio of clinker production to cement production in various provinces.

## Technical Validation

### Uncertainty analysis

In general, there are two primary sources of uncertainty for cement-related emissions. One comes from the uncertainty of activity data; the other one pertains to the uncertainty of emission factors^78^. The uncertainty of the activity data is further comprised of uncertainties from the clinker output data of the cement industry, the clinker output data of enterprises, and the under-counted amount of kiln dust. The clinker output may be under-reported by local cement industry authorities. The clinker output provided by enterprises may contain technical errors of clinker weighing. The under-counted amount of kiln dust refers to the kiln ash lost during limestone burning, which contains calcium oxide after the decomposition of calcium carbonate. The uncertainty of emission factors comes from the sampling error of *MgO* and *CaO* contents in clinker and the error of chemical analysis of clinker conducted by enterprises. It may also come from the error of *MgO* and *CaO* contents in raw materials brought into the final calculation^20,28^.

The IPCC Good Practice Guide suggests that the uncertainty of plant-level clinker data is typically around 1–2%^79^.This study uses the median value of 1.5%. The industry statistics of clinker output are generally completed by the China Building Materials Quantitative Economic Supervision Committee, whose uncertainties are unclear. In *the 2005 China Greenhouse Gas Emissions Inventory Study*, the level of uncertainty was set at 5%, and the uncertainty caused by the correction coefficient of the kiln dust is 0.3%^28^. Based on this, the combined uncertainty of raw clinker production data is 6.8%. For those estimated clinker production data, this study temporarily set a higher uncertainty at 10%. The uncertainty in our clinker production data for all years are determined in this way.

The IPCC Good Practice Guide sets the uncertainty of chemical analysis at 1–2%^79^. This study uses the upper limit value of the range as the 2005 uncertainty of chemical analysis, i.e. 2%. The sampling error is about 0.2%^29^. Hence, the combined uncertainty of actual clinker emission factor of Chinese provinces is 2.2% in 2005 and 1994. The estimated clinker emission factor of the province by the regional average value is likely to have a higher uncertainty, and we set the uncertainty level at 4% in 2005. The 2005 clinker emission factors, which were derived from the 2005 China Greenhouse Gas Emission Inventory study, are used in the calculation of process-related emissions during 2000–2019. The 1994 clinker emission factors, derived from the 1994 China Greenhouse Gas Emission Inventory study, are used in the calculation of process-related emission during 1993–1999. The clinker emission factors are likely to vary with years, so that the uncertainty of the clinker emission factor in other years will be higher than the values in 2005 and 1994. Taking the period 2000–2019 as an example, the content of calcium carbonate and magnesium carbonate in the limestone for the production of clinker in various provinces are likely different with the value in 2005. We assume that it change gradually. This means that the closer the year to 2005, the smaller the changes in the clinker emission factor and hence the uncertainty; as the year interval increases, the corresponding uncertainty grows. Therefore, for the uncertainties of clinker emission factors in other years of period 2000–2019, we assume that they are based on the uncertainties in 2005 and increase every five years according to the rule: increase by 0.1 for each year in the first five years, 0.2 for each year in the second five years and 0.3 for each year in the third five years.

**Fig. 6:**
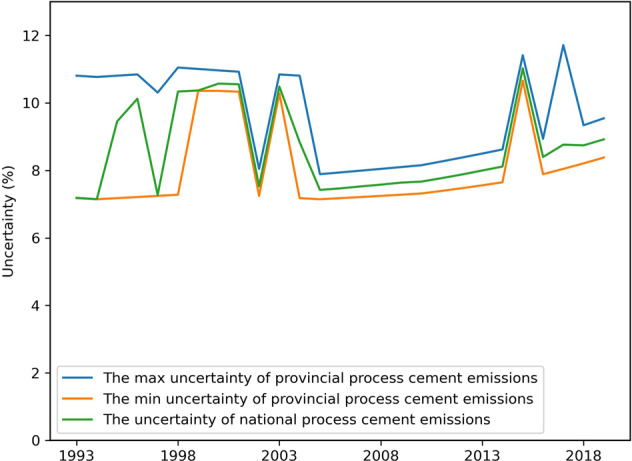
The max and min uncertainty in provinces and the uncertainty of national process cement emissions during 1993–2019 in China by the error propagation method.

This study uses the error propagation method (Approach 1) recommended by the IPCC to determine uncertainties of provincial process-related CO_2_ emissions of cement production. According to the multiplication relationship of clinker production data and clinker emission factor, the combined uncertainty of each province’s carbon dioxide emissions is 7.1–7.9% in 2005. During 1993–2019, the combined uncertainties of provincial carbon dioxide emissions range from 7.1% to 11.7%. The max and min of them by years are shown in Fig. 6.

For the combined uncertainty of the process carbon dioxide emissions from China’s total cement production over the years, this study use two methods, including the error propagation method and the Monte Carlo method. First, the combined uncertainty of the national clinker production is calculated with the average uncertainty of provincial clinker production with the weight of provincial clinker production. The combined uncertainty of national average clinker emission factor is calculated with the uncertainty of provincial clinker emission factor with the weight of provincial clinker production. Then the combined uncertainty of national process-related cement emission is around 7.1–11.0% with the error propagation method during 1993–2019, as shown in Fig. 6. Second, under the normal distribution for the national clinker production and clinker emission factor, we employ the Monte Carlo method to calculate the combined uncertainty for the national process emission of cement industry. Based on determining the combined uncertainty of the national clinker production and the average clinker factor, we respectively calculated the standard deviation of the normal distribution patterns. Then we conduct random sampling on both the activity data and emission factors for 200,000 times and get 200,000 estimations on the national process-related CO_2_ emissions in the cement industry. The uncertainty range, therefore, was 95% confidential intervals of the estimations. The Monte Carlo simulation was conducted in Python3.8. The uncertainties of national process-related cement emission through the Monte Carlo simulations range from 7.131% to 11.018% during 1993–2019. As introduced above in the Emission coefficient subsection, there are some variations of average national clinker emission factor in the several clinker-based sources: IPCC, *1994 China National Greenhouse Gas Inventory Study* (NGGI1994), *2005 China National Greenhouse Gas Inventory Study* (NGGI2005), CSI and Shan *et al*.^21^. The Coefficient of Variation (CV, the standard deviation divided by the mean) by these institutions is 3.2%. In the 1994 China Greenhouse Gas Emission Inventory study, the combined uncertainty of national process-related CO_2_ emission in cement industry is about 6.6–8.3%. The calculated uncertainties of this study by the two methods are 7.1%, which are close to the calculated uncertainty of the 1994 China Greenhouse Gas Emission Inventory study. In addition to the above uncertainties of clinker emission factors and activity data, some other uncertainties should also be considered when using the datasets. For example, the carbon absorption effect in the use of cement has been neglected in the calculation, which also creates a certain degree of uncertainty. There may be differences between the China Cement Association and the National Bureau of Statistics in the statistical methods of cement production and clinker production. This difference in data sources may also increase the uncertainty of the calculation results.

### Comparison with previous cement emission estimates

**Fig. 7:**
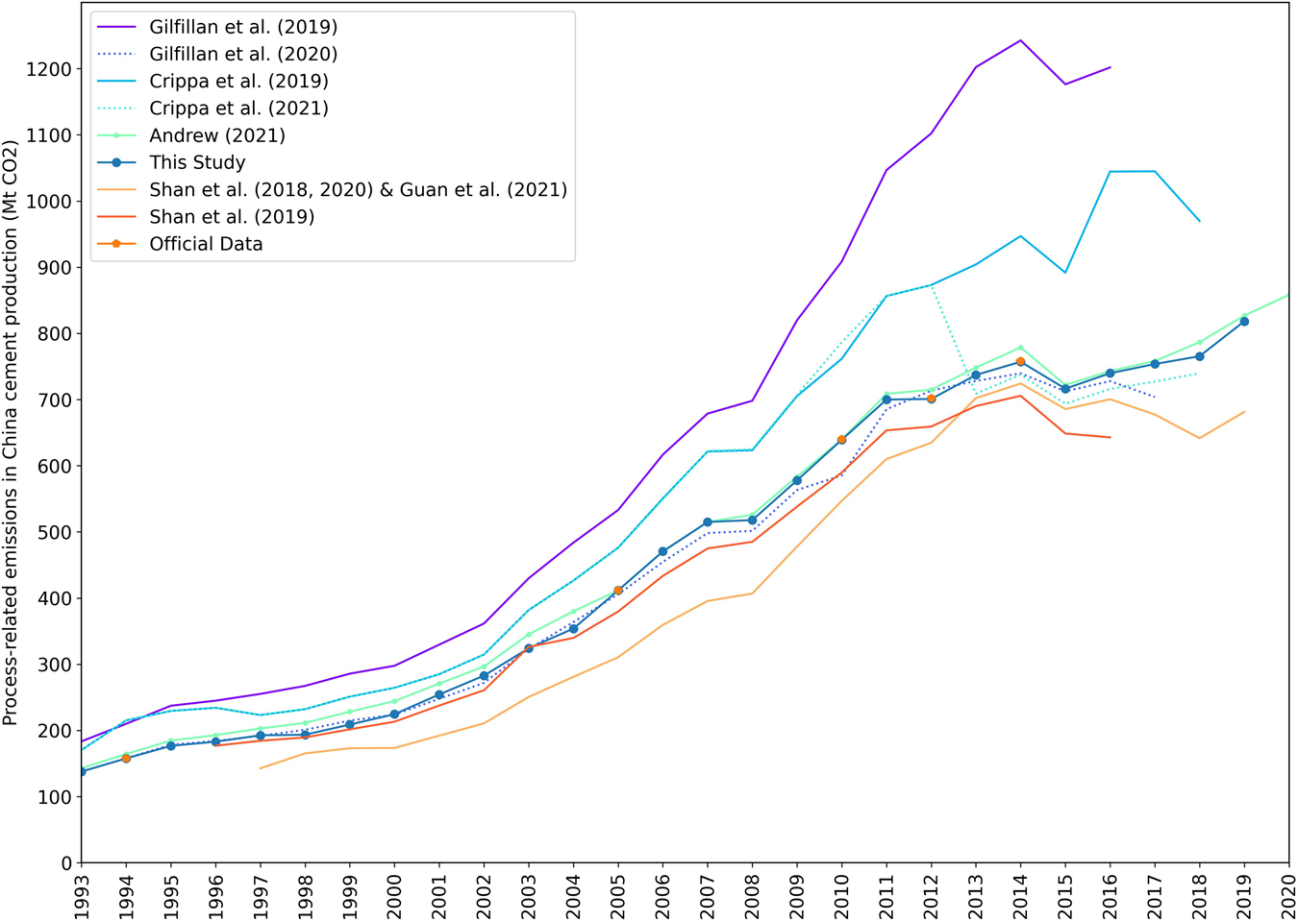
Process emissions from China’s cement production, 1993–2019. Also shown are estimates from CDIAC (Gilfillan *et al*.^12,80^), EDGAR v5.0 (Crippa *et al*.^13^), EDGAR v6.0 (Crippa *et al*.^14^), GCB (Andrew^11^, data version 210723), CEADs with the cement-based method (Shan *et al*.^16^,^18^; Guan *et al*.^19^) and clinker-based method (Shan *et al*.^21^).

To verify the cement emissions data given in this study, we compared our results with estimates of other professional institutes or research groups, as shown in Fig. 7. It illustrates that our estimates for cement-related emissions of China are lying in the middle range, close to the official estimates, with less than 1% difference.

According to the first and second national communications submitted by Chinese officials to the United Nations Framework Convention on Climate Change (UNFCCC)^2^, process emissions from the cement production process in Mainland China in 1994 and 2005 were 157.8 Mt CO_2_^6^ and 411.7 Mt CO_2_^7^, respectively. In the third national communication, the first and the second Biennial Update Report, the process emissions of non-metallic mineral products and the clinker production were reported, but the process emissions of cement production were not directly reported. In 2010, 2012 and 2014, China’s cement clinker production was 1.18875 Gt clinker^10^, 1.3392 Gt clinker^9^ and 1.4865 Gt clinker^8^, respectively. The 2005 National Greenhouse Gas Inventory reported that the national average carbon emission factor for clinker was 0.5383. Like Andrew (2019), we use this clinker emission coefficient to estimate the total cement process carbon emissions in Mainland China^2^. In 2010, 2012 and 2014, its process emission were be roughly 639.9 Mt CO_2_, 701.9 Mt CO_2_ and 758.3 Mt CO_2_, respectively.

Our estimation results suggest that China process-related cement CO_2_ emissions have been likely overestimated by the old version data of CDIAC and EDGAR. For example, in the year 2012, the old version of CDIAC overestimated 55% of the emissions and EDGAR overestimated by 22%. However, due to the lower emission factors of cement and clinker, the cement-based results and clinker-based results of CEADs have underestimated China’s cement-related CO_2_ emissions by 10% and 7%, respectively. The new version data of CDIAC by Gilfillan *et al*.^12^ show much lower cement process emission in China than its old version data of CDIAC by Gilfillan *et al*.^12,80^. In 2010, 2012 and 2014, the gap between CDIAC’s new version of China’s cement process data and the results of China’s greenhouse gas inventory was −8.5%, 1.6%, and 2.5%, respectively. Since 2013, EDGAR 6.0^14^ has shown much lower values than EDGAR v5.0^13^ for China’s process-related cement CO_2_ emissions because of an adjustment acknowledging the lower clinker ratio. Based on the comparisons (Fig. 7), only the estimated values of GCB^11^ and the new version data of CDIAC (Gilfillan *et al*.)^12^ are close to the results of this study, and the differences between GCB and this study are generally within 2.5% in the last ten years.

**Fig. 8:**
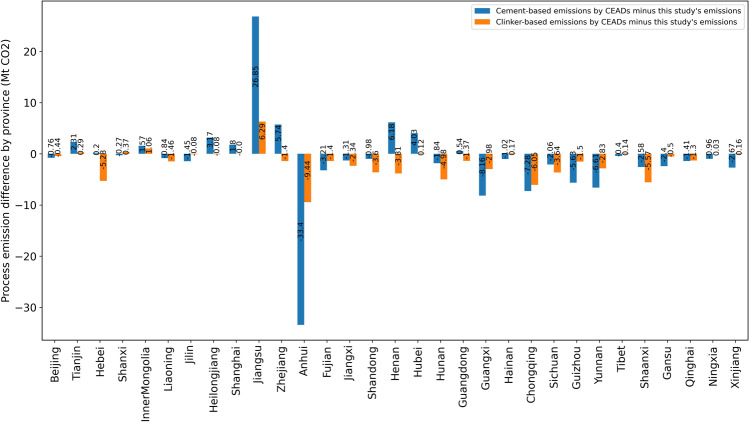
The provincial difference of process emissions between this study and CEADs in 2014 (Mt CO_2_).

In previous studies, only CEADs have provided provincial-level CO_2_ emission estimates for the cement industry with both the cement-based method and clinker-based method. The process emission difference between this study and CEADs by provinces in 2014 are shown in Fig. 8. The difference between the results of this article and the two methods of CEADs varies from province to province. In Jiangsu, the cement-based and clinker-based estimates by CEADs is 26.9 Mt CO_2_ and 6.3 Mt CO_2_ higher than this study’s estimates, respectively. In Anhui, the cement-based and clinker-based estimates by CEADs are 33.4 Mt CO_2_ and 9.4 Mt CO_2_ lower than this study’s estimates, respectively. In Henan, the cement-based estimate is 6.2 Mt CO_2_ higher than this study’s estimates, but the clinker-based estimate is 3.8 Mt CO_2_ lower than this study’s estimates. Our provincial-level clinker emission factor estimation provides the latest and longest-term emission inventory of China and its provinces, and is an important supplement to existing emission estimates and official emission inventories. Also, the two methods of CEADs adopted the national average emission factor, which cannot reflect provincial differences in the cement manufacturing process and cement–clinker ratios across China.

### Limitations

Our dataset has the following limitations: first, despite we applied the clinker emission factors of various provinces in China, the emission factor of the year 1994 and 2005 was assumed to be representative for the time interval of 1993–1999 and 2000–2019 respectively. Considering the differences in clinker production technology in different years, variations in annual emission factors are expected. In future studies, the provincial clinker emission factors of different years, if possible, should be applied to achieve a more accurate emission inventory estimation of the cement production process. Second, due to the lack of clinker data for the years 1995–1996, 1998–2001 and 2003–2004, we assumed that the clinker-to-cement ratio is constant, so that we used the averages of the clinker-cement ratio of the previous and next years, and the annual cement data of the previous years to estimate the clinker data. Third, this study uses the standard territorial principle in estimating the emissions of each province. We use the perspective of administrative boundaries to calculate the CO_2_ emissions of the cement production process, without considering the impact of the transfer of cement and cement clinker between different provinces from the perspective of final consumption.

## Data Availability

The data file with XLSX format are accessible on ScienceDB. No additional code is used during the calculation of provincial process CO_2_ emissions from China cement production. The installation of Microsoft Office or WPS Office is recommended to manage the data and reproduce the study results.
